# 56 nm Wide-Band Tunable Q-Switched Erbium Doped Fiber Laser with Tungsten Ditelluride (WTe_2_) Saturable Absorber

**DOI:** 10.1038/s41598-020-66664-9

**Published:** 2020-06-17

**Authors:** Harith Ahmad, Hissah Saedoon Albaqawi, Norazriena Yusoff, Chong Wu Yi

**Affiliations:** 1 Photonics Research Centre, University of Malaya, 50603 Kuala Lumpur, Malaysia; 2 Physics Department, Faculty of Science, University of Malaya, 50603 Kuala Lumpur, Malaysia; 3 Visiting Professor at the Department of Physics, Faculty of Science and Technology, Airlangga University, Surabaya, 60115 Indonesia

**Keywords:** Physics, Applied physics, Optical physics

## Abstract

A wide-band and tunable Q-switched erbium-doped fiber (EDF) laser operating at 1560.5 nm with a tungsten ditelluride (WTe_2_) saturable absorber (SA) is demonstrated. The semi-metallic nature of WTe_2_ as well as its small band gap and excellent nonlinear optical properties make it an excellent SA material. The laser cavity uses an 89.5 cm long EDF, pumped by a 980 nm laser diode as the linear gain while the WTe_2_ based SA generates the pulsed output. The WTe_2_ based SA has a modulation depth, non-saturable loss and saturation intensity of about 21.4%, 78.6%, and 0.35 kW/cm^2^ respectively. Stable pulses with a maximum repetition rate of 55.56 kHz, narrowest pulse width of 1.77 µs and highest pulse energy of 18.09 nJ are obtained at the maximum pump power of 244.5 mW. A 56 nm tuning range is obtained in the laser cavity, and the output is observed having a signal to noise ratio (SNR) of 48.5 dB. The demonstrated laser has potential for use in a large number of photonics applications.

## Introduction

Pulsed lasers are highly desirable laser sources that are typically realized in the form of mode-locked^1^ and Q-switched lasers^2^ that can be further classified as either active^3^ or passive^4^ systems. Active pulsed laser is accomplished through the use of acousto-optic^5^ and electro-optic modulators^6^ among other technique to generate pulses. This approach gives significant control over the various parameters of the generated pulses, but at the cost of a bulky and expensive setup. Normally, passive pulse laser can be typically achieved through the use of saturable absorbers (SAs)^7,8^. This approach, while providing less control over the output parameters of the generated pulses, is advantageous in that it allows for compact and cost-effective fiber laser cavities to be designed^9^. Topological insulators (TIs) such as bismuth selenide (Bi_2_Se_3_)^10^, bismuth telluride (Bi_2_Te_3_)^11^, black phosphorus^12,13^, perovskite^14^, as well as carbon-based materials such as carbon nanotubes (CNTs)^15^ and graphene^16^ are amongst the various materials that have shown great potential as SAs in order to produce pulses in erbium-doped fiber (EDF) laser cavities. This is because the afore-mentioned groups of materials exhibits a larger nonlinear-optical response^17^ as well as exceptional optical properties that include a large absorption coefficient, low defect density and long carrier lifetimes^18^. Besides that, the thin 2D structure of these materials give it an advantage in terms of unique photonic, magnetic, and electronic properties that is crucial for pulsed laser generation^19^.

Q-switched pulse generation has long been the focus of research efforts due to its advantageous intrinsic characteristics that include long pulses with higher pulse energies and durations^20^, which is highly desirable for applications in material processing, remote sensing, range finding, and medicine^21–25^. Furthermore, Q-switching is easier to induce as compared to mode-locking^26^, which would require delicate balancing between the dispersion and nonlinearities in the laser cavity^27^. As such, Q-switching is generally the preferred method of obtaining pulses in a laser cavity. Recently, researchers have focused their attention towards the exploration of transition metal dichalcogenide (TMD) group of materials in generating Q-switched pulses at broadband wavelengths. As SAs, TMD have significant advantages that include strong absorption^28^, high nonlinear optical response, optical fiber compatibility and ease of fabrication^29^. Luo *et al*.^30^ demonstrated passively Q-switched Ytterbium (Yb), Erbium (Er) and Thulium (Tm) fiber lasers using a molybdenum disulfide (MoS_2_) film as an SA, and their findings show that few-layer MoS_2_ films have significant potential as broadband SAs operating at the near to mid-infrared regions. Furthermore, Zhang *et al*.^31^ demonstrated the use of tungsten disulfide (WS_2_) to passively Q-switch EDF and Yb-fiber laser cavities. The WS_2_ based SA is capable of generating Q-switched pulses with microsecond durations and kilohertz repetition rates at lasing wavelengths of 1030 and 1558 nm, demonstrating the potential for WS_2_ as an SA in ultrafast photonic applications.

Tungsten ditelluride (WTe_2_) is another member of the TMD family and is a unique material as it is associated to a rising class of Weyl semimetals. This makes them highly promising for future applications as electronic, spintronic, and optoelectronic devices^32,33^ and also as a potential candidate for quantum spin Hall insulator materials^34^. Even though WTe_2_ belongs to the TMD family, its uniqueness arises from the additional structural distortion caused by the W atoms forming zigzag chains in a quasi-one-dimensional arrangement^35^. Furthermore, the small overlap between the valence band and conduction band of WTe_2_ results in an almost gapless band; as small as 0.7 eV^36^ compared to other TMD materials which typically have band gaps of more than 1 eV^37^. As such, WTe_2_ is more suitable for applications in near-infrared systems such as photodetectors, communications devices and in the area of ultrafast optics. WTe_2_ was also been found to have unique characteristics such as a high unsaturated magneto-resistance (MR) as well as good superconducting behaviour while under high pressure^38^ which makes WTe_2_ quite attractive for nanoelectronic applications^39^. In addition to that, the very fast relaxation of photocarriers makes WTe_2_ suitable for generating Q-switched pulses^38^. In a study conducted by Wang *et. al*.^40^, an ultrafast pulse was successful generated in a mode-locked thulium-doped fiber laser through the use of magnetron-sputtering deposited WTe_2_ as an SA. Stable soliton pulses with pulse durations of 1.25 ps and average output powers of 39.9 mW were obtained at a central wavelength of 1915.5 nm. Similarly, Koo *et. al*.^41^, developed passively mode-locked ultrafast lasers at 1556.2 nm using defective, bulk-structured WTe_2_ microflakes as an SA. They discovered that the structural dimensionality does not critically influence the saturable absorption performance of WTe_2_.

In this work, WTe_2_ is demonstrated as a wideband SA in a passively Q-switched EDFL operating at the 1.5 μm region. The WTe_2_ SA has a modulation depth of 21.4% and saturation intensity of ~0.35 kW/cm^2^. Stable Q-switched pulses are obtained at a central lasing wavelength of 1560.5 nm, which can be tuned over a range of 56 nm from 1522 nm to 1578 nm. The proposed SA would have significant benefits and potential to be used in various applications.

## Characterization of a Tungsten Ditelluride (WTe2) - Based SA

**Figure 1:**
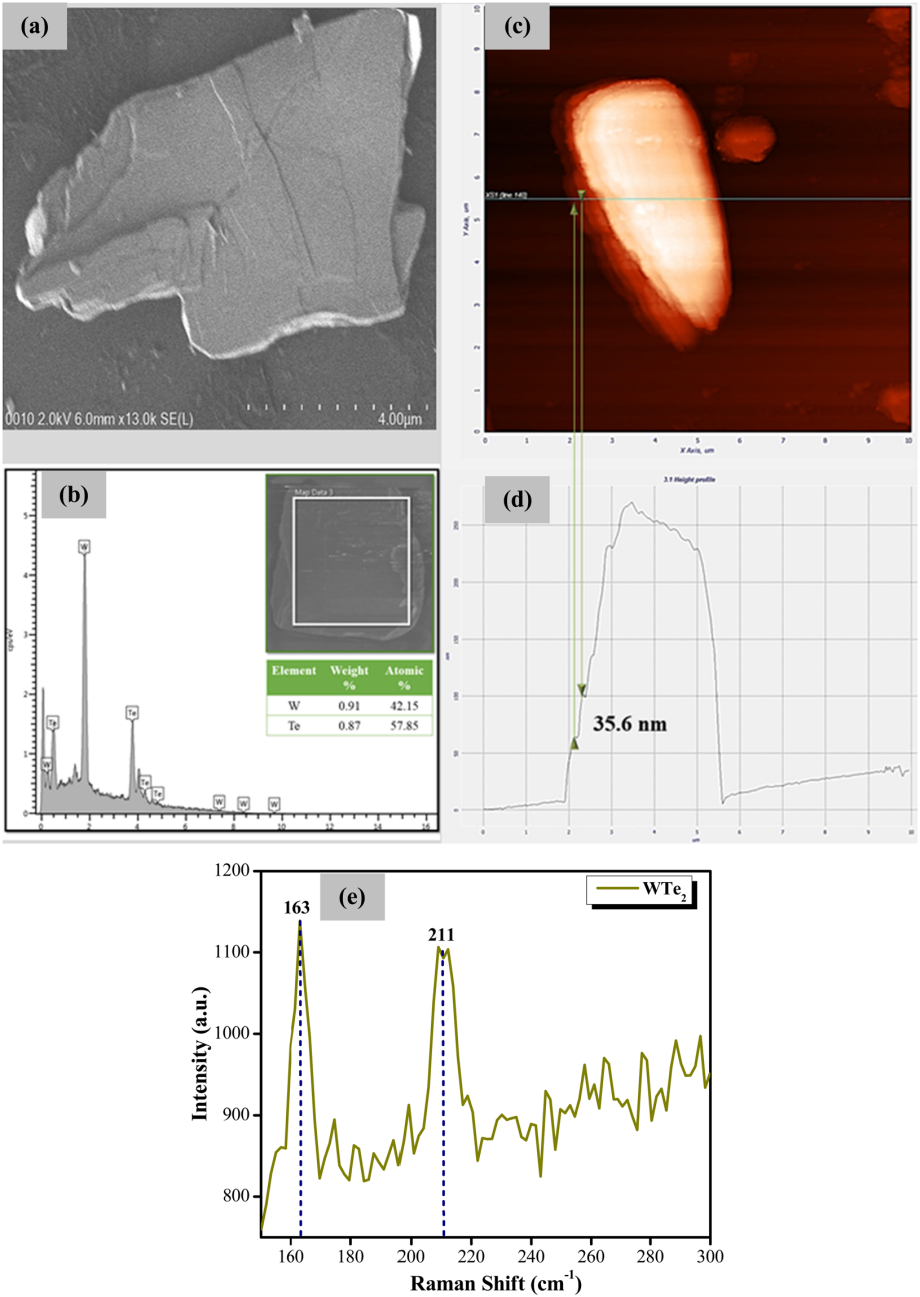
(**a**) FESEM image, (**b**) EDX spectrum, (**c**) AFM image, (**d**) Lateral height measurement, and (**e**) Raman spectrum for few layers WTe_2_.

Figure 1(a) shows the morphology of the WTe_2_ layer as obtained from a Hitachi SU8220 field-emission scanning electron microscope (FESEM) under x13k magnification. From the figure, it can be seen that the WTe_2_ nanoparticles have a flake-like morphology, and tend to stack together to form thicker flakes. Figure 1(b) shows the energy dispersive X-ray (EDX) profile of the WTe_2_ sample, with signal peaks associated only with the tungsten (W) and tellurium (Te) elements observed. This shows both the formation of WTe_2_ sample as well as its purity. The inset of Fig. 1(b) shows the area of the WTe_2_ sample from which the scan was obtained, as well as the weight and atomic percentage of the two elements which are 0.91% and 42. 15%, for W and 0.87% and 57.85% for Te respectively.

An NT MDT atomic force microscope (AFM) is used to measure the thickness of the WTe_2_ film. The location of the WTe_2_ film’s surface that was chosen for the thickness measurement is denoted by the blue line. The obtained AFM image is shown in Fig. 1(c), and it can be seen that the WTe_2_ sample used in this work consists of about six individual layers stacked together. Figure 1(d) gives the height profile of WTe_2_ film projected within the blue line. Six steps can be counted on the height profile, which gives the estimated thickness of the WTe_2_ film to be six layers. The green line represents the position of one WTe_2_ layer, with the thickness of a single layer estimated to be about 35.6 nm. As such, the overall thickness of the sample is estimated to be 213.6 nm. Further characterization of the WTe_2_ sample is also carried out using a Renishaw inVia Raman microscope linked with a 532 nm line from a doubled Nd:YAG laser as the excitation source. As displayed in Fig. 1(e), two distinct Raman peaks are seen in the Raman spectrum of WTe_2_. The peaks located at 163 and 211 cm^−1^ can be assigned to the in-plane A_1_^7^ and A_1_^9^ modes of WTe_2_, respectively^42,43^.

**Figure 2:**
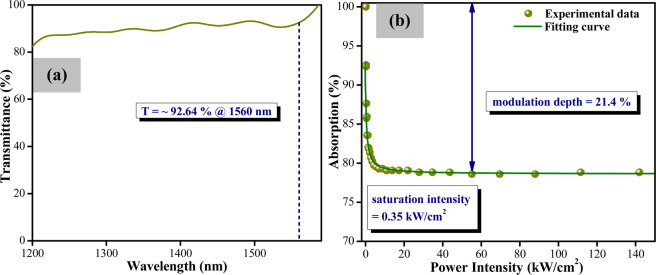
(**a**) Linear optical transmission and (**b**) Nonlinear optical absorption of WTe_2_-PVA film.

The SA assembly is formed by sandwiching a small piece of the WTe_2_-PVA between two optical fiber patchcords. A small amount of index matching gel is placed on the surface of the optical fiber patchcord, on which the WTe_2_-PVA piece is then placed. Using a fiber adaptor, another patchcord is joined to the first, thus forming the SA assembly. A white light source is linked to the SA as to obtain the linear optical transmission characteristics of the WTe_2_ film. The optical spectrum is observed from 1200 nm to 1600 nm using a Yokogawa AQ6370C optical spectrum analyser (OSA) with an average transmission (T) of 92.64% observed at 1560 nm as in Fig. 2(a). The nonlinear optical absorption characteristic of the WTe_2_ SA are investigated using the balanced twin detector technique, with a Menlo Systems ELMO femtosecond erbium laser with a 100 MHz repetition rate and 2.88 ps pulse width at 1564 nm serving as the signal source. The obtained data is inserted into the saturation model equation^44^:

$$\alpha (I)=\frac{{\alpha }_{s}}{1+I/{I}_{sat}}+{\alpha }_{ns}$$

where *α*_*s*_, *I*, *I*_*sat*_, and *α*_*ns*_ is refer to the modulation depth, input intensity, saturation intensity, and non-saturable loss respectively. The relative parameters for the WTe_2_ SA are obtained based from the resulting fitted curve as given in Fig. 2(b), with the modulation depth, saturation intensity and non-saturable loss of the WTe_2_ SA being ~ 21.4%, 0.35 kW/cm^2^, and 78.6%, respectively. The insertion loss of the WTe_2_ SA is measured to be approximately 0.31 dB.

## Experimental Setup

**Figure 3:**
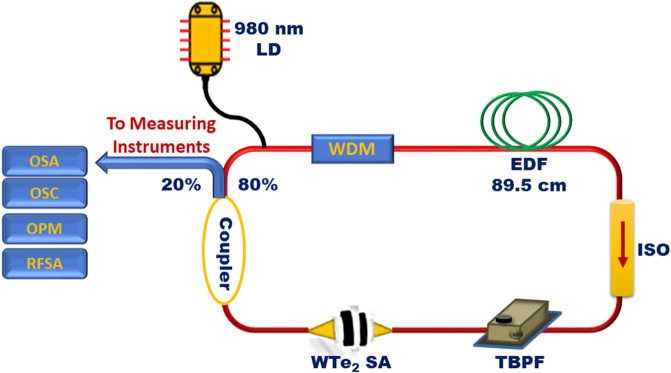
Schematic illustration of the experimental setup for generating EDF laser with the assistance of WTe_2_ SA. (LD: laser diode; WDM: wavelength division multiplexer; EDF: Erbium-doped fiber; ISO: isolator; WTe_2_ SA: Tungsten ditelluride saturable absorber; OSA: optical spectrum analyzer; OSC: oscilloscope; OPM: optical power meter; RFSA: radio frequency spectrum analyzer; TBPF: tunable optical bandpass filter).

Figure 3 shows the schematic of the passively Q-switched ring cavity EDF laser. The laser cavity uses a 980 nm laser diode (LD) as the pump source with a maximum output power of 244.5 mW and injected into the cavity via the 980 nm port of a 980/1550 nm wavelength-division multiplexer (WDM). The output of the WDM is connected to a 89.5 cm long EDF which has a dopant concentration, absorption, mode field diameter, and numerical aperture of 2000 ppm, 16 dB/m at 1530 nm, 9.5 µm at 1550 nm and 0.13, respectively. The EDF serves as the gain media, and is connected to an optical isolator as well as an optical tunable bandpass filter (TBPF) with a tuning range of 80 nm and a resolution of 1 nm. The signal now reaches the WTe_2_ based SA, and an 80:20 optical coupler is used to obtain 20% of the signal. The 80% port of the coupler is connected to the 1550 nm port of the WDM, thereby closing the optical cavity. Polarization controllers (PCs) are not included in the design of the cavity as Q-switched pulses can be observed immediately with the incorporation of the WTe_2_ based SA into the laser cavity and without any further optimization of the propagating signal. Therefore, adding a PC would not improve the performance of the cavity, and will instead induce additional insertion losses to the cavity.

## Results and Discussion

**Figure 4:**
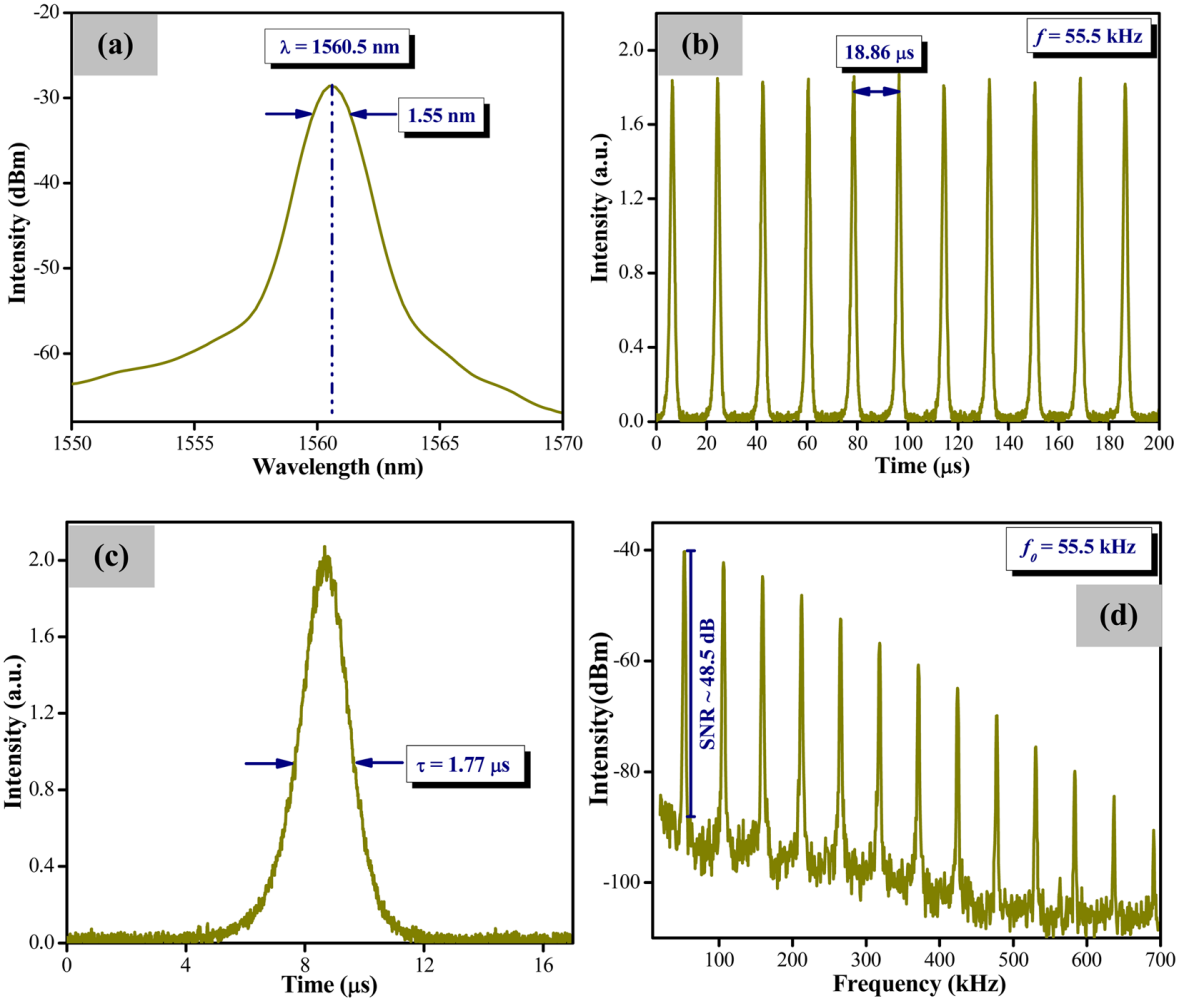
(**a**) Optical spectrum of the laser output, (**b**) pulse train (**c**) single pulse profile and (**d**) RF spectrum. All measurements are taken at a pump power of 244.5 mW:.

When operating without the WTe_2_ SA, no pulsed outputs can be detected, thus confirming that the generation of pulses is due solely to the SA and not arising from other optical phenomena. With the WTe_2_ SA in the cavity, continuous wave (CW) operation is obtained at a threshold pump power of 77.43 mW while Q-switching begins at a threshold pump power of 124.9 mW. Pulsing is observed to continue steadily until the maximum pump power of 244.5 mW is reached. The pulse characteristics of the Q-switched EDF laser at a pump power of 244.5 mW are shown in Fig. 4. The optical spectrum of the pulses is given in Fig. 4(a), where the central wavelength is determined to be 1560.5 nm with a 3-dB band width of 1.55 nm. The oscilloscope trace of the Q-switched pulses is given in Fig. 4(b), whereby the generated pulses have a peak-to-peak pulse interval of 18.86 μs that corresponds to a repetition rate of 55.56 kHz. In Fig. 4(c), the single pulse profile has a full width at half maximum (FWHM) duration of 1.77 μs. Based on the RF spectrum, a frequency of 55.56 kHz is observed in Fig. 4(d), with the pulses haveing an average signal-to-noise ratio (SNR) of about 48.5 dB. This indicates that the generated Q-switched pulses are highly stable, and comparable to that of other similar systems^45,46^.

**Figure 5:**
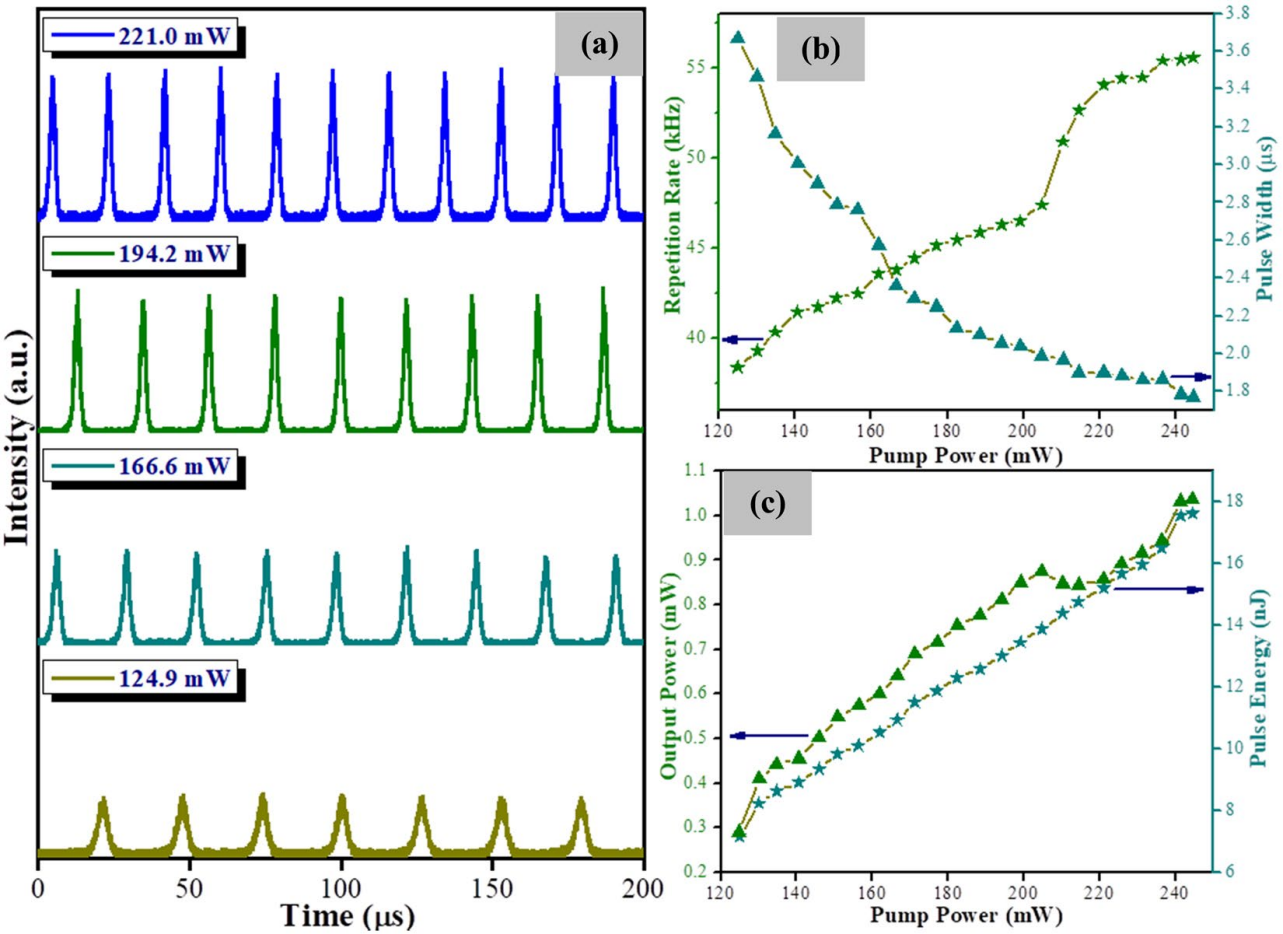
(**a**) Passively Q-switched pulse trains at the pump powers of 124.9 mW, 166.6 mW, 194.2 mW, and 221 mW, respectively, (**b**) repetition rate and pulse width and (**c**) output power and pulse energy.

Figure 5(a) shows the oscilloscope traces of the output pulses obtained at four different pump powers. It can be seen from the figure that the repetition rate increases while the spacing between pulses decreases as the pump power is increased. These trends are characteristic of Q-switching operation. The repetition rate and pulse width as a function of pump power is given in Fig. 5(b) and shows the repetition rate increasing from 38.39 kHz to 55.56 kHz when the pump power is increased from 124.9 mW to 244.5 mW. At the same time, the pulse width decreases from 3.67 μs to 1.77 μs, with a sharp decrease in the pulse width before a pump power of 236.5 mW and a slower decrease after it. The slower decrease of the pulse width at the higher pump powers is attributed to the change in the saturable-absorption property of the WTe_2_ SA from parasitic continuous waves that can partially bleach it^47^. As shown in Fig. 5(c), the variation of the output power and pulse energy against the pump power shows a generally rising trend, rising from 0.28 mW to 1.01 mW and 7.31 nJ to 18.09 nJ, respectively as the pump power is pushed to the maximum. However, as can be seen from the figure, a decrease in the average output power is observed at a pump power of 204.8 mW, before the average output power continues to rise normally against the increasing pump power. This sudden decrease in power is a result of a kink in the output power of the LD as the drive current increases. This causes the output power from the fiber laser to drop slightly, then continue to increase normally as the drive current increases. Another possibility as put forward by Adel^48^ is that when the drive current increased, this causes an increase in the temperature of the laser diode, and shifts the lasing wavelength away from the peak absorbing wavelength of 974 nm. This reduces the absorption occurring in the EDF, thus lowering the output power of the fiber laser. Further increasing the drive current of the LD will increase the pump power and cause the output power of the fiber laser to increase in tandem, as would be expected.

**Figure 6:**
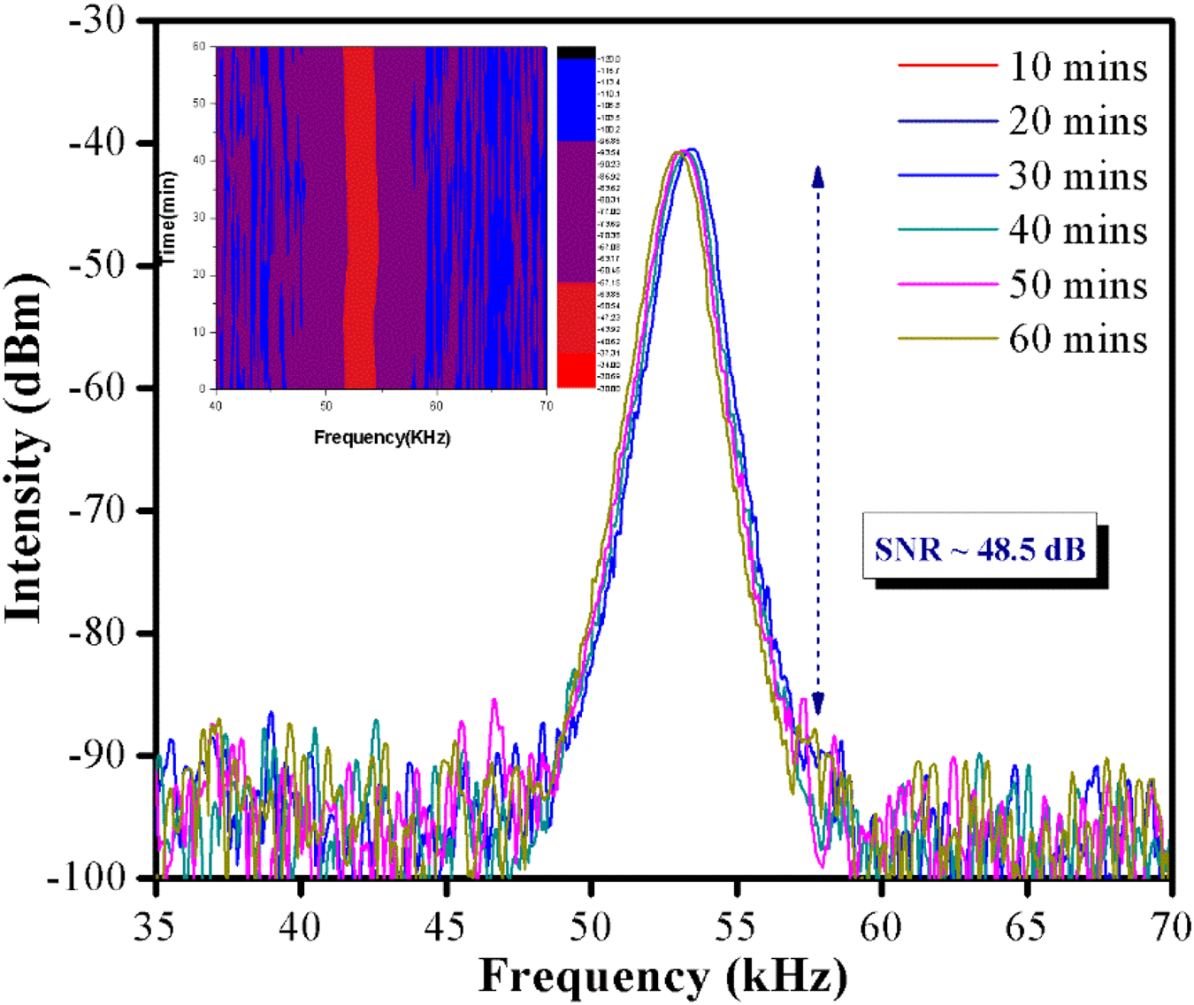
Stability performance of the passively Q-switched EDF laser within 60 minutes recorded using RFSA at a constant pump power of 221 mW.

Figure 6 gives the RF spectra as captured over a span of 60 minutes at a pump power of 221 mW. No significant changes in the SNR value over the time period can be observed, with the average SNR value being ~48.5 dB. Based on the inset of Fig. 6, no frequency drifting can be observed with the frequency remaining constant at about ~53.8 kHz throughout the observation period. These results indicate the high stability of the proposed laser and also that there is no significant degradation of the WTe_2_ SA’s performance throughout its operation period.

**Figure 7:**
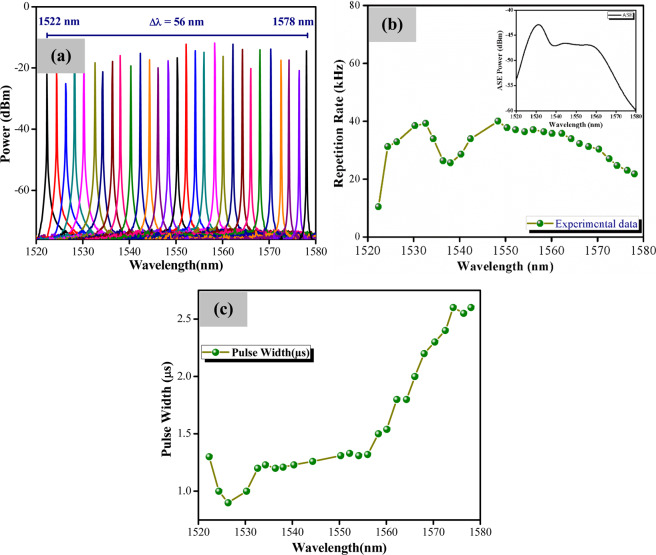
(**a**) Superimposed optical spectra of the tunable EDF laser at different wavelengths, (**b**) the repetition rate of Q-switching against the tunable lasing wavelength at constant pump power of 194.2 mW (Inset: ASE spectrum), and (**c**) the pulse width versus lasing wavelength.

Figure 7(a) shows the optical spectra of the tunable wavelength output obtained at a constant pump power of 194.2 mW. From the figure, no Q-switching operation can be observed below the wavelength of 1522 nm or beyond the wavelength of 1578 nm, giving the laser a tuning wavelength of 56 nm. Figure 7(b) shows the repetition rates against different wavelengths, and from the figure it can be seen that there is a gradual increase in the repetition rate as the wavelength is tuned from 1522 to 1532 nm. In general, the high repetition rate at the larger gain region of the cavity is due to lower cavity losses. This happens as a result of the more rapid bleaching of the SA which is due to faster population inversion/depletion rates^49^. Above a wavelength of the 1532 nm, the variation of repetition rate trend follows the amplified spontaneous emission (ASE) spectrum of the laser, as the inset of Fig. 7(b), from which the EDF’s gain profile is obtained^50^. The variation of the pulse width of the Q-switched laser output over the same wavelength tuning range is given in Fig. 7(c). From the figure, it can be seen that the pulse width increases from 1.3 μs to 2.6 μs over the increasing wavelength range. This is attributed to the different wavelength regions experiencing different gains, and as such variations in the repetition rate and pulse width that correspond to the gain curve of the cavity.

A comparison of the passively Q-switched fiber laser in this work against other similar systems using different SAs is given in Table 1. From the table, it can be seen that the proposed laser of this work using the WTe_2_-PVA film based SA generates Q-switched pulses with the narrowest pulse width. Furthermore, the threshold pump power for Q-switching to occur in this system is lower compared other similar systems^29^. Overall, the output performance of the proposed laser system is comparable and at some points is better than that in previous reports, thus confirming the applicability of WTe_2_ as an SA for Q-switched pulse generation at the C-band region. The excellent photoresponse behaviour possessed by tellurium (Te)^51^ allows for good pulsed laser performance to the realized.

**Table 1:** Passively Q-switched fiber laser operating at 1.5 μm by different SAs.

Saturable absorber	Operation wavelength (nm)	Tunable wavelength range (nm)	Pump power range (mW)	Pulse width (μs)	Repetition rate (kHz)	Maximum pulse energy (nJ)	Ref.
MoSe_2_ - PVA	1560	—	570–720	4.04–6.506	60.724–66.847	369.5	^52^
WSe_2_ - PVA	1560	—	280–720	4.063–9.182	46.281–85.365	484.8	^52^
MoWSe_2_	1554	—	99–245	6.80-1.90	26–48	11.80	^53^
MoS_2_ – PVA	1565	—	17.40–134.30	23.20-5.40	6.50–27	63.20	^30^
MoS_2_– PVA	1560	1519.6–1567.7	18.9–227.1	26.7-3.3	8.77–43.47	160	^54^
LPE Chitosan/MoS_2_	1561.5	1510–1580	135.4–280.5	1.68 - 1.02	57.3–79.4	43.69	^55^
WSSe	1568.4	1530–1570	89.07–280.5	4.16-2.6	27.52–61.81	7.31	^56^
MoSe_2_-PVA	1562	—	22.4–102.0	59.1-30.4	16.9–32.8	57.9	^57^
WTe_2_	1531	—	212–630	~2.3-0.583	144.7–240	58.625	^58^
SnS	1560	—	275–500	—	36.36–65.19	—	^59^
BP	1988	—	—	1.78	19.25	7840	^60^
WTe_2_ - PVA	1560. 5	1522–1578	124.9–244.5	3.67-1.77	38.39–55.56	18.09	This work

MoS_e_ = Molybdenum disulfide, MoSe_2_ = Molybdenum diselenide, WSe_2_ = Tungsten diselenide, MoWSe_2_ = Molybdenum tungsten diselenide, WSSe = Tungsten sulfide selenide, SnS = Tin Sulfide, BP = Black phosphorus.

## Methods

### Preparation of a Tungsten Ditelluride (WTe2) - Based SA

Solution casting is used to fabricate the WTe_2_ film with a polyvinyl alcohol (PVA) polymer thin film host. The WTe_2_ solution is purchased from 2D Semiconductors at 99.99% purity while the PVA at MW~31,000 powder is obtained from Sigma Aldrich. Approximately 100 mg of the PVA powder is slowly added into 10 ml of deionized water (DIW) at 60 °C and stirred continuously for 2 hours using a magnetic stirrer. A homogeneous WTe_2_ solution is obtained by treating the purchased WTe_2_ solution with a bath sonicator for a period of 30 minutes. Approximately 2 mL of the homogeneous 1 mg/mL WTe_2_ solution is added drop by drop into a beaker containing 8 mL of the 10 mg/mL PVA solution while stirring. The mixture is stirred continuously for another 15 minutes at 60 °C. Finally, the mixture is poured into a glass petri dish and heated in an oven for 2 hours at 60 °C, and the WTe_2_/PVA film is carefully removed from the petri dish after being allowed to cool down to room temperature.

### Laser characterization

Analysis of the sample signal is done using the Yokogawa AQ6370C OSA as well as a Yokogawa DLM2054 oscilloscope (OSC) with a 1 GHz photodetector. An Anritsu MS2683A radio frequency spectrum analyzer (RFSA) and a Thorlabs optical power meter (OPM) are used to monitor the output spectra for further analysis.

## Conclusion

In this work, a broadband WTe_2_ based SA is demonstrated for the passive generation of Q-switched pulses in the C-band region. A stable Q-switched output is achieved at a threshold pump power of 124.9 mW with a central wavelength of 1560.5 nm. By increasing the pumping power from 124.9 mW to 244.5 mW, the repetition rate rises from 38.39 kHz to 55.56 kHz while the pulse width decreases from 3.67 µs to 1.77 µs. The proposed laser system exhibits wide-band tunability of up to 56 nm from 1522 nm to 1578 nm, and is highly stable with no significant fluctuation in frequency observed over an operation period of 60 minutes with average SNR values of 48.5 dB. The obtained experimental results imply that WTe_2_ has a great potential as SA and the proposed laser system can be used as a pulse tunable laser source for various optical telecommunications and measurement applications.
